# Loss of interleukin-1 beta is not protective in the lupus-prone NZM2328 mouse model

**DOI:** 10.3389/fimmu.2023.1162799

**Published:** 2023-05-16

**Authors:** Shannon N. Loftus, Jianhua Liu, Celine C. Berthier, Johann E. Gudjonsson, Mehrnaz Gharaee-Kermani, Lam C. Tsoi, J. Michelle Kahlenberg

**Affiliations:** ^1^Division of Rheumatology, Department of Internal Medicine, University of Michigan, Ann Arbor, MI, United States; ^2^Graduate Program in Immunology, University of Michigan, Ann Arbor, MI, United States; ^3^Division of Nephrology, Department of Internal Medicine, University of Michigan, Ann Arbor, MI, United States; ^4^Department of Dermatology, University of Michigan, Ann Arbor, MI, United States; ^5^Department of Computational Medicine and Bioinformatics, University of Michigan, Ann Arbor, MI, United States; ^6^Department of Biostatistics, University of Michigan, Ann Arbor, MI, United States

**Keywords:** lupus, nephritis, interleukin-1beta (IL-1ß), inflammasome, proteinuria

## Abstract

Aberrant activation of the innate immune system is a known driver of lupus pathogenesis. Inhibition of the inflammasome and its downstream signaling components in murine models of lupus has been shown to reduce the severity of disease. Interleukin-1 beta (IL-1β) is a proinflammatory cytokine released from cells following inflammasome activation. Here, we examine how loss of IL-1β affects disease severity in the lupus-prone NZM2328 mouse model. We observed a sex-biased increase in immune complex deposition in the kidneys of female mice in the absence of IL-1β that corresponds to worsened proteinuria. Loss of IL-1β did not result in changes in overall survival, anti-dsDNA autoantibody production, or renal immune cell infiltration. RNA-sequencing analysis identified upregulation of TNF and IL-17 signaling pathways specifically in females lacking IL-1β. Increases in these signaling pathways were also found in female patients with lupus nephritis, suggesting clinical relevance for upregulation of these pathways. Together, these data suggest that inhibition of the inflammasome or its downstream elements that block IL-1β signaling may need to be approached with caution in SLE, especially in patients with renal involvement to prevent potential disease exacerbation.

## 1 Introduction

Systemic lupus erythematosus (SLE) is an autoimmune disease involving aberrant immune responses, production of autoantibodies, and multiorgan system involvement. The etiology of SLE is multifactorial, likely involving a complex interplay between genetic risk factors and environmental triggers (1). Dysregulation of several innate and adaptive immune signaling pathways contributes to the inflammation characteristic of the disease. Multiple cytokines have been implicated in the pathogenesis and progression of SLE including B-cell activating factor (BAFF), type I interferons (IFN), and members of the interleukin-1 (IL-1) superfamily (2–4). While drugs targeting BAFF (belimumab) and the type I IFN receptor (anifrolumab) have been approved for treatment of SLE, the specific roles of IL-1 cytokines in SLE have remained more elusive (5, 6).

IL-1 beta (IL-1β) and IL-18, members of the IL-1 superfamily, are synthesized as inactive precursor molecules that are processed to their mature, biologically active form following activation of the inflammasome. Enhanced inflammasome activation is observed in lupus macrophages and monocytes, and studies suggest that inhibition of inflammasome signaling attenuates disease severity in murine models of lupus (7–10). Specifically, suppression of inflammasome activation through blockade of caspase-1 or the NLRP3 inflammasome has been shown to reduce severity of lupus nephritis (LN) and decrease autoantibody production (8, 10).

Most studies to date have focused on the role of the inflammasome complex in the modulation of disease activity, and many of them have only highlighted correlative associations between IL-1β/IL-18 and disease severity (8, 11). As a result, the specific roles and mechanisms of these individual cytokines in SLE are incompletely understood. Given that IL-1β can trigger a broad range of responses that drive systemic inflammation and exacerbate damage during chronic disease, we hypothesized that inhibition of this cytokine would limit disease severity in a lupus-prone mouse model.

In this study, we examined how loss of IL-1β modulates disease in the NZM2328 lupus-prone mouse model. We observed no differences in overall survival or autoantibody production between NZM and NZM-*Il1b*^-/-^ mice. Surprisingly, we identified a female-specific increase in immune complex deposition in the kidneys in the absence of IL-1β. Consistent with these results, female NZM-*Il1b*^-/-^ mice also had increased proteinuria compared with NZM controls. RNA-sequencing analysis of the kidneys identified sex-specific differences in TNF and IL-17 signaling pathways that are also observed in female LN patients. These results demonstrate an unexpected potentially protective role for IL-1β in LN in a sex-biased manner. This suggests that IL-1β blockade could have unintended consequences for disease progression in LN patients.

## 2 Results

### 2.1 Deletion of IL-1β does not improve survival of lupus-prone mice

Lupus-prone NZM2328 (NZM) mice spontaneously develop lupus-like characteristics including autoantibody production and glomerulonephritis with females developing more severe manifestations at an earlier age of onset compared with males (12). To investigate the role of IL-1β in driving disease pathology in this model, we used NZM mice with a homozygous deletion of *Il1b* (NZM-*Il1b*^-/-^) (13).

**Figure 1:**
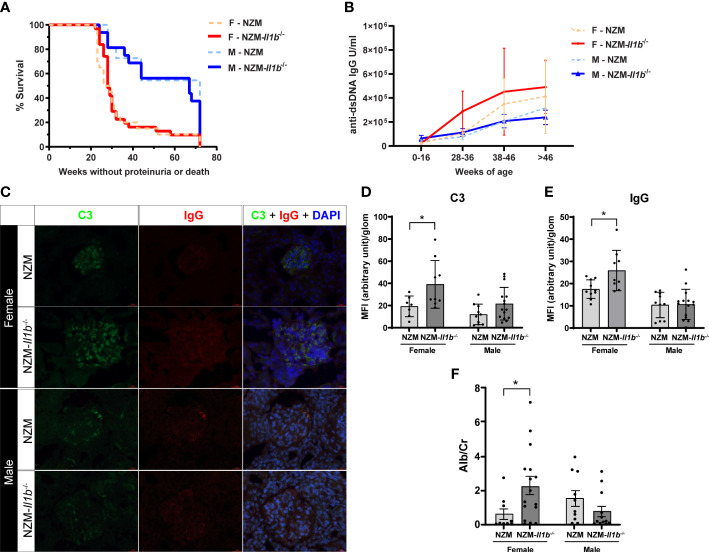
Absence of IL-1β has no effect on survival but increases renal immune complex deposition **(A)** Survival of female (F) and male (M) NZM and NZM-Il1b-/- mice was monitored for 72 weeks or until mice became moribund following development of proteinuria (n = 11–31). **(B)** Levels of anti-dsDNA IgG were measured in the serum at the indicated age ranges by ELISA (n = 3–20 per age range). **(C)** Representative images of immunofluorescent microscopy of renal cortex containing glomeruli. Green = FITC-C3, red = Texas Red-IgG, blue = DAPI. Levels of **(D)** C3 and **(E)** IgG were quantified using a semiautomated analysis program (n = 7–15). **(F)** The ratio of microalbumin (Alb) to creatinine (Cr) was assessed in the urine at time of death as a measure of proteinuria (n = 10–16). Data analyzed by the log-rank test, unpaired t test, or Mann–Whitney test. *<0.05.

We first examined how loss of IL-1β impacted the overall survival of both male and female mice. While disease progression was much slower in male mice, as expected, there were no differences in survival outcomes within the sexes between NZM and NZM-*Il1b*^-/-^ mice (**Figure 1A**). Next, we measured production of anti-dsDNA antibodies, a hallmark of disease that generally begins by 5 months of age in female NZM mice and is delayed by approximately 4 weeks in males (12). Serum levels of anti-dsDNA IgG increased over time in both male and female mice but were not significantly different between NZM and NZM-*Il1b*^-/-^ (**Figure 1B**).

### 2.2 Absence of IL-1β results in increased immune complex deposition in the kidneys

LN is characterized by the deposition of immune complexes within the kidney that can promote renal inflammation and damage contributing to development of proteinuria. We thus quantified immune complex deposition in the kidneys. Surprisingly, we observed a significantly increased staining of both C3 (*p = 0.0397*) and IgG (*p = 0.0176*) in the kidneys of female NZM-*Il1b*^-/-^ mice compared with NZM controls, indicating increased immune complex deposition. No significant difference was observed between male NZM and NZM-*Il1b*^-/-^ mice (C3: *p = 0.0798*; IgG: *p = 0.9238*) (**Figures 1C–E**). We then examined the urine to see if this increase in immune complexes translated into any meaningful change in proteinuria. In line with the immune complex staining, female NZM-*Il1b*^-/-^ mice had increased albumin-to-creatinine ratios (*p = 0.0196*), indicative of damage to the filtration barrier, compared with their NZM counterparts, whereas no significant difference was observed for males at this timepoint (*p = 0.1593*) (**Figure 1F**).

Together, these data suggest that whereas IL-1β does not affect overall survival or autoantibody production in lupus-prone mice, it may play an important sex-specific protective role in the kidney by limiting immune complex deposition and resultant proteinuria.

### 2.3 Loss of IL-1β does not change immune cell infiltration into kidneys

**Figure 2:**
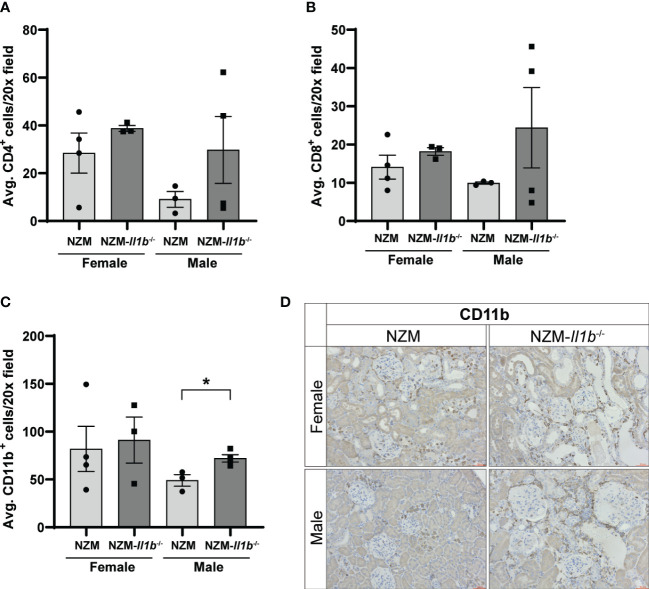
Loss of IL-1β does not change immune cell infiltration into kidneys. Quantification of **(A)** CD4+, **(B)** CD8+, and **(C)** CD11b+ cells in the kidney per ×20 field averaged across three images each (n = 3–4). **(D)** Representative images of kidneys stained with CD11b. Data analyzed by unpaired t tests. *<0.05.

LN is characterized by an inflammatory cascade involving immune cell infiltration into the kidneys followed by production of proinflammatory cytokines and chemokines that contribute to kidney damage (14). To determine if gross differences in immune cell infiltration were observed in the female NZM-*Il1b*^-/-^ kidneys, we examined the presence of CD4^+^, CD8^+^, CD11b^+^, and CD11c^+^ cells by immunostaining. While CD4^+^ (female: *p = 0.3513*; male: *p = 0.2725*) and CD8^+^ (female: *p = 0.3306*; male: *p = 0.2966*) T cells, as well as CD11b^+^ myeloid cells (female: *p = 0.7987*), were detected in the kidneys, there were no significant differences in the numbers of these cells between female NZM and NZM-*Il1b*^-/-^ mice (**Figures 2A–D**; **Figures S1A, B**). Male NZM-*Il1b*^-/-^ mice did have significantly more CD11b^+^ cells compared with NZM (*p = 0.0195*) (**Figures 2C, D**). CD11c^+^ dendritic cells were not identified in the kidneys (**Figure S1C**). Based on these data, we see similar populations of immune cells in the kidneys of these mice.

### 2.4 IL-17 and TNF signaling pathways are activated in females in the absence of IL-1β

**Figure 3:**
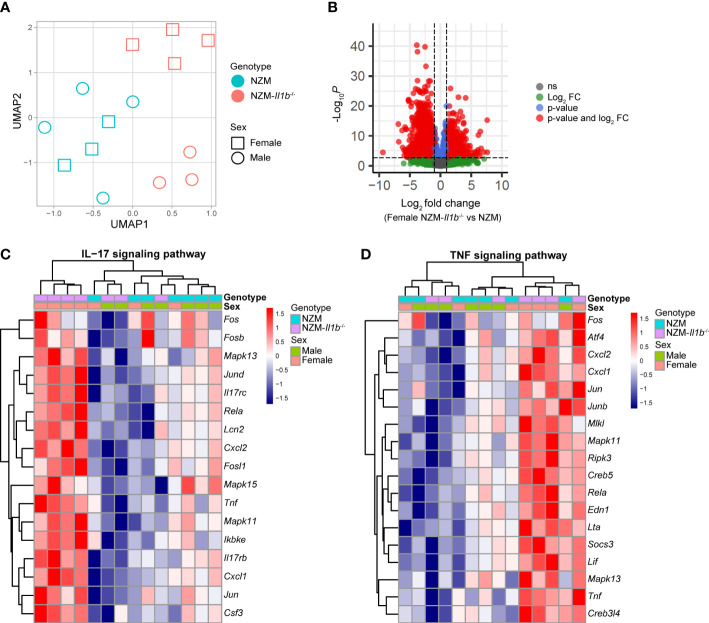
Sex-biased increase in IL-17 and TNF signaling pathway gene expression in the absence of IL-1β. **(A)** Dimension reduction (UMAP) plot for RNA-seq kidney samples. **(B)** Volcano plot to show summary statistics of the differential expression analysis comparing NZM-Il1b-/- vs. NZM in female mice. **(C-D)** Heatmaps illustrating standardized expressions of genes participating in the **(C)** IL-17 and **(D)** TNF signaling pathways.

To better understand the impact of IL-1β deletion on lupus kidneys, genome-wide transcriptome analysis using RNA-sequencing (RNA-seq) was performed and transcriptional differences in the kidneys of male and female NZM and NZM-*Il1b*^-/-^ mice were examined. As expected, *Il1b* transcripts were significantly downregulated in the NZM-*Il1b*-/- kidneys compared with NZM in both male and female mice (female: FC = 0.42, p = 0.014; male: FC = 0.17, p = 6.75 × 10^-7^). We performed uniform manifold approximation and projection (UMAP) for dimension reduction on the transcriptome data, and we highlighted that male and female mice separated from each other only in NZM-*Il1b*^-/-^ but not NZM kidney samples, emphasizing the sex bias seen in the mouse model (**Figure 3A**). Indeed, when comparing differentially expressed genes (DEGs) between NZM and NZM-*Il1b*^-/-^ kidneys (using FDR <0.01 and fold change of 2), we identified 1,046 and 1,992 genes that were up- and downregulated, respectively, for female mice (**Figure 3B**), whereas for male there were only 112 up and 382 down genes.

Pathway analysis from those DEGs highlighted the TNF (*p = 7.59 × 10^-5^*) and IL-17 signaling (*p = 1.21 × 10^-6^*) pathways among the top uniquely regulated pathways (**Figure S2**) in the NZM-*Il1b^-/-^* mice.

Heatmaps of the genes involved in the modulated TNF and IL-17 pathways confirmed a robust upregulation of TNF and IL-17-related genes in kidneys of female NZM-*Il1b*^-/-^ mice compared with the kidneys of male NZM-*Il1b*^-/-^ mice (**Figures 3C, D**). Ingenuity Pathway Analysis was used to predict upstream regulators for the DEGs identified in the kidneys of female and male NZM-*IL1b*^-/-^ vs. NZM mice (**Table S1**). TNF was identified as the top upstream regulator in female mice. These data suggest that enhancement of the IL-17 and TNF signaling pathways occurs in a sex-biased manner in the absence of IL-1β signaling and that TNF may be a driving differentiator of this signal.

**Figure 4:**
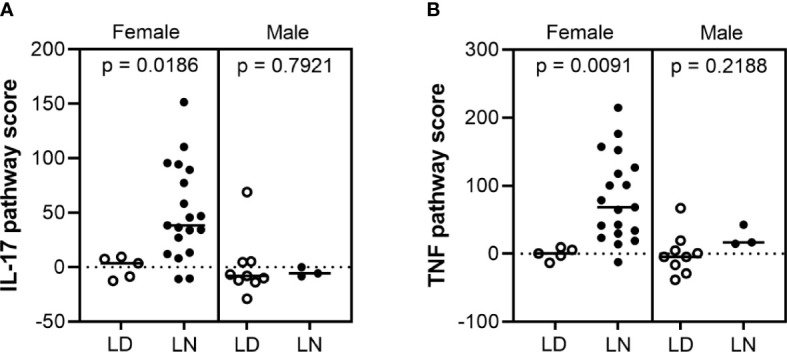
IL-17 and TNF pathway scores increased in female lupus nephritis patients. Microarray gene expression analysis of **(A)** IL-17 and **(B)** TNF pathway scores in the glomerular compartment of human lupus nephritis **(LN)** and healthy living donor **(LD)** biopsies: IL-17 and TNF signaling pathway scores were significantly higher in female LN (n = 19) compared to LD controls (n = 5) (p = 0.0186 and 0.091, respectively), but not in males (n = 9 LD and 3 LN) (p = 0.7921 and 0.2188, respectively).

To determine whether sex-biased regulation of IL-17 and TNF pathways has clinical relevance in LN patients, we studied available gene expression data of microdissected renal biopsies from healthy living donors and patients with class III and/or IV LN (**Table S2**). IL-17 and TNF signaling pathway signature scores (see Methods) were significantly elevated in the glomeruli of female LN patients compared with living donors (*p = 0.0186* and *0.0091*, respectively), but not in males (*p = 0.7921* and *0.2188*, respectively) (**Figures 4A, B**). Those results were validated in a second independent LN patient cohort (**Table S3**, **Figures S3A, B**). Interestingly, these signatures are not seen in the tubule compartments from the same patients, highlighting that this may be a glomerular-specific pathology (**Figures S3C-F**). This further supports our murine data suggesting involvement of these pathways in promoting sex-biased differences in kidney disease in the absence of IL-1β.

## 3 Discussion

In this study, we uncovered a novel sex-biased protective role for IL-1β in limiting renal pathology in the lupus-prone NZM2328 mouse model. While loss of IL-1β did not significantly alter survival or autoantibody production, it did increase glomerular immune complex deposition and worsen proteinuria in a female-specific manner. We observed no differences in immune cell infiltration into the kidneys to explain these differences. RNA-seq analysis identified upregulation of genes involved in IL-17 and TNF signaling pathways in female, but not male, NZM-*Il1b*^-/-^ compared with NZM mice. Importantly, upregulation of these signaling pathways was also observed in female LN patients compared with healthy controls, suggesting that activation of IL-17 and TNF may have relevance in the sex bias in human disease and should be further investigated.

Our data support an unexpected role for IL-1β in limiting renal damage in LN. Previous studies have offered differing interpretations of the role of IL-1β in SLE. Indeed, inhibition of the activating caspase and inflammasome complexes has protective effects on LN in many murine models. Both IL-1β and IL-18 are secreted following inflammasome activation; however, the individual contributions of these cytokines to SLE are incompletely understood. Serum levels of IL-18 are elevated in SLE patients and correlate with disease activity and organ damage (11, 15, 16). Furthermore, in murine lupus models, IL-18 serum levels correlate with LN severity (8, 17). Therefore, inflammasome activation of IL-18 may be responsible for driving pathogenic effects in SLE instead of IL-1β.

Some studies have identified increased expression of IL-1β in the serum of SLE patients (18, 19) and in lupus-prone mouse models (20). Our data suggest that IL-1β is not critical for nephritis progression but may in fact have a protective effect that is revealed when its function is inhibited in isolation (as opposed to full inflammasome complex targeting). This may also be evidence that increased levels of IL-1β in lupus is an extraneous consequence of inflammasome activation and not actually indicative of a pathogenic effect. Indeed, recent studies support a protective effect for signaling through the IL-1 receptor in podocytes during glomerular diseases (21, 22). Our data exhibit more proteinuria, but not inflammation, in the absence of *Il1b*, which may suggest a role for protective IL-1 podocyte signaling in our model as well. Furthermore, in our human data, the IL-17 and TNF signatures are only increased in the glomeruli, not the tubules, of female LN patients suggesting that this might be a podocyte-associated effect of IL-1β. Lastly, IL-1β has been shown to induce the expression of CD16 (FcγRIII), a receptor involved in immune complex clearance, suggesting that the worsened renal pathology in our model may, in part, result from reduced ability to clear immune complexes (23, 24).

We have identified an upregulation of IL-17 signaling pathways in NZM-*Il1b*-/- mice. This is surprising, as IL-1β plays a critical role in differentiation of Th17 cells (25, 26), which are the canonical producers of IL-17. While it is seemingly contradictory that loss of IL-1β would result in the increase of a signaling pathway for which IL-1β is known to be important, there are other cell types that produce IL-17 that may explain this. Populations of DN T cells are expanded in SLE patients and in some lupus-prone mouse models (27, 28), and these cells have been shown to be major producers of IL-17 (29). Furthermore, these IL-17-producing DN T cells have been identified in kidney biopsies from patients with LN (29). In a murine model of crescentic glomerulonephritis, the primary source of IL-17 in the kidney was shown to change over the course of the disease (30). Later in disease, most IL-17 was produced by Th17 cells. However, at early time points, γδ T cells were the major producers of IL-17 with additional contributions from DN T cells and NKT cells. These IL-17^+^ γδ T cells were dependent on renal dendritic cell-derived IL-23 (another cytokine reported to be highly expressed in human SLE sera (31) and the transcription factor RORγt. Absence of γδ T cells in this model resulted in less neutrophil and macrophage infiltration into the kidney as well as reduced levels of serum creatinine, suggesting a pathogenic role for the IL-17-producing γδ T cells (30). Intriguingly, and relevant to our work, IL-17 production from γδ T cells was not dependent on IL-1β (30). This suggests a potential mechanism by which loss of IL-1β, an important cytokine for Th17 cells, can still result in enhanced IL-17 signaling.

Our study has a few limitations to note. First, the study was completed comparing homozygous *Il1b-/-* mice and did not include heterozygotes to examine gene dosage effects. Second, we chose a survival timepoint of 72 weeks, which may have blunted our ability to see differences in survival, especially in the male mice. If we had a cohort of mice at an earlier timepoint, we may have also noted differences in the timing of onset of immune complexes in the *Il1b*-deficient compared with the NZM mice. Finally, our human nephritis cohort was from a European consortium, which included only Caucasian patients, which limits our ability to extend our human data to patients of other ethnic and racial backgrounds without further study.

Enthusiasm for IL-1 inhibition in SLE has remained low until late. A small clinical trial of anakinra, a recombinant version of IL-1Ra, was conducted with four SLE patients with severe, treatment-refractory polyarthritis (32). While subjective improvements were observed in patients, one patient had an arthritic flare, and studies in larger cohorts of patients were not conducted. No signs of SLE flares in the kidneys were identified, but enrolled patients did not have a history of renal disease and specific clinical markers of lupus nephritis were not measured. Recently, other studies of IL-1 blockade using anakinra have shown promise in improving SLE-associated recurrent fevers, pericarditis, and macrophage activation syndrome, highlighting potential benefits for specific acute manifestations of lupus (33–36). Our data, however, suggests that chronic inhibition of IL-1 signaling, especially in patients with renal involvement, should be approached with caution to potentially prevent aggravation of disease.

Elevated levels of type I IFNs are a prominent feature of SLE that plays an important role in driving development and progression of lupus nephritis (37). Previous work suggests that inflammasome activation negatively regulates the expression of type I IFNs through IL-1β-mediated signaling in the context of malaria infection (38). In our study, we did not identify differential expression of type I IFN signaling pathways between the NZM-*Il1b*^-/-^ and NZM kidneys. This lack of difference could suggest that regulatory mechanisms in an infection setting may differ from those in an autoimmune setting in which inflammatory pathways, including type I IFN pathways, are already highly activated and dysregulated. Prior work indicates that females mount higher type I IFN responses that make them more prone to autoimmune diseases (39). This suggests that, perhaps, loss of the repressive effects of IL-1β nudges the type I IFN-dependent nephritis even further. This could partially explain the sexual dimorphism that is seen in these mice.

In summary, we have described an unexpected inflammatory regulatory role for IL-1β in renal injury in female NZM2328 mice. In its absence, mice develop worsened proteinuria and immune complex deposition in a sex-specific manner. We hypothesize that the more highly dysregulated inflammatory environment present in female NZM mice is needed for this sex-biased response. Further research into the specific mechanisms at play in our model is needed; until then, caution regarding the use of inflammasome and IL-1 inhibitors in human lupus (especially in patients with lupus nephritis) should be considered.

## 4 Materials and methods

### 4.1 Mice

New Zealand Mixed (NZM) 2328 lupus-prone mice were a gift from Dr. Chaim Jacob, University of Southern California. NZM-*Il1b*^-/-^ mice (NZM2328 mice lacking functional IL-1β) were generated through the University of Michigan Transgenic Animal Model Core, as previously described (13). All mice were bred and housed in specific pathogen-free facilities at the University of Michigan and treated in accordance with our University of Michigan IACUC-approved protocol. Survival studies were conducted for 72 weeks in male and female mice or until mice became moribund following development of proteinuria. Mice were monitored for development of lupus *via* weekly urine collection starting at 20 weeks of age. Blood was sampled *via* saphenous vein bleed every other week. At euthanasia, terminal bleeding was performed *via* cardiac puncture and tissues were harvested.

### 4.2 Quantification of autoantibodies

Anti-dsDNA IgG levels were quantified in the serum using the Mouse Anti-dsDNA IgG ELISA Kit (Alpha Diagnostics International), according to the manufacturer’s protocols.

### 4.3 Proteinuria analysis

Urine samples were assessed for microalbumin using the mouse Albuwell M Kit (Exocell, Philadelphia, PA) and creatinine using the mouse QuantiChrom™ Creatinine Assay Kit (BioAssay Systems; Hayward, CA), both according to the manufacturer’s protocols. Mircoalbumin-to-creatinine ratios were calculated to estimate 24-h urinary protein excretion.

### 4.4 Immune complex deposition scoring

Glomerular immune complex deposition was quantified on frozen kidney sections *via* staining for C3 and IgG deposition, as previously described (40). Briefly, sections were stained with FITC-conjugated anti-C3 (1:250; Immunology Consultants Laboratory, Portland, OR) and Texas-Red-conjugated anti-IgG (1:250; Invitrogen, Waltham, MA) for 1 h at 4°C. DAPI was used to visualize DNA. Glomerular immune complex staining was quantified using FIJI ImageJ and a semiautomated looping program to capture fluorescence within a user-defined area designed by the BRCF Microscopy Core at the University of Michigan.

### 4.5 Immunohistochemistry

For detection of immune cells in mouse kidney, formalin-fixed, paraffin-embedded sections were heated at 60°C for 1 h, deparaffinized, rehydrated, and heated at 100°C for 20 min in Retrievagen A (pH 6.0) (for CD11c; BD Biosciences, Franklin Lakes, NJ) or Tris–EDTA buffer (pH 9.0) (for CD11b, CD4, and CD8) for antigen retrieval. Slides were washed, treated with 3% hydrogen peroxide in PBS for 5 min, blocked in goat serum for 1 h, and incubated with Recombinant Anti-CD11c antibody [EPR21826] (1:50; ab219799), Recombinant Anti-CD4 antibody [EPR19514] (1:500; ab183685), Recombinant Anti-CD8 alpha antibody [EPR21769] (1:2,000; ab217344), or Recombinant Anti-CD11b antibody [EPR1344] (1:4,000; ab133357) (Abcam, Boston, MA) overnight at 4°C. Isotype controls (#3900; Cell Signaling Technology, Danvers, MA) were stained in parallel with each set of slides. All slides were incubated with biotinylated goat anti-rabbit IgG secondary antibody (1:200; Vector Laboratories, Newark, California), followed by incubation with VECTASTAIN Elite ABC Reagent (Vector Laboratories, Newark, CA) and detection with 3,3′-diaminobenzidine (BD, Franklin Lakes, NJ) under a light microscope. Slides were counterstained with hematoxylin, dehydrated, and mounted. Images were acquired using a Zeiss microscope (Zeiss, Oberkochen, Germany) at indicated magnifications. Positive cells were quantified by manually counting the # of positive cells (brown) averaged for five 20× fields of view.

### 4.6 RNA-sequencing

Kidney RNA was isolated *via* Zymo Direct-zol RNA isolation kit, and libraries were generated using standard poly-A prep kits (New England Biolabs) with the assistance of the U-M Advanced Genomics Core. An average of 61 million read pairs were obtained per sample *via* NextSeq 6000. Paired-end reads (151 bp for each end) were generated for the RNA-seq experiments. After quality control and adapter trimming, we conducted read alignment (41) and gene quantification (42) using mm10 and Gencode vM18, respectively. DESeq2 was used for read normalization and modeling (43). Only genes with on average greater than one read per sample were used in subsequent analysis.

### 4.7 Human renal biopsy samples and calculation of IL17 and TNF signaling pathway signature scores

Gene expression analyses of glomeruli and tubulointerstitial compartments from the European cDNA Bank (ERCB) human renal biopsies were used, as previously described (44, 45). In brief, the discovery cohort included pretransplant healthy living donors (LD; 5 females, 9 males for glomeruli; 6 females, 3 males for tubules) and patients’ demographic characteristics representative of lupus nephritis disease (LN; 19 females, 3 males for glomeruli; 6 females, 3 males for tubules) and WHO class III and/or IV. The validation cohort included 5 LD (4 females, 1 male for glomeruli and tubules) and LN patients (10 female, 3 male for glomeruli; 18 female, 6 male for tubules), also WHO class III and/or IV (ref: www.Nephroseq.org).

IL-17 and TNF pathway scores were calculated using the algorithm described by Feng et al. (46), and as previously published (47), using sex-matched LD controls. The IL-17 and TNF signaling pathway genes were extracted from KEGG (https://www.genome.jp/entry/pathway+hsa04657 and https://www.genome.jp/entry/hsa04668). The IL-17 pathway score was calculated for glomeruli using 77 and 80 of the 94 genes that were expressed in the discovery and validation datasets, respectively, and for tubules using 74 and 82 of the 94 genes that were expressed in the discovery and validation datasets, respectively (**Table S4**). The TNF pathway score was calculated for glomeruli using 99 and 108 of the 112 genes that were expressed in the discovery and validation datasets, respectively, and for tubules using 95 and 109 of the 112 genes that were expressed in the discovery and validation datasets, respectively (**Table S4**).

### 4.8 Upstream regulator analyses

Ingenuity Pathway Analysis (IPA) software was used to identify potential upstream transcriptional regulators, which may be involved in the regulation of the genes differentially expressed in NZM-*Il1b*^-/-^ compared to NZM mice.

### 4.9 Statistical analysis

Data (**Figures 1**, **2**, **4**, **Supplementary Figure 3**) were graphed and statistics were performed using GraphPad Prism 9. Data are presented as the mean ± SEM (**Figures 1**, **2**) or with median line (**Figure 4**, **Figure S3**). For comparisons between two groups, unpaired two-tailed t tests or Mann–Whitney tests were used. For survival studies, log-rank testing was used. p-values <0.05 were considered as statistically significant.

## Data availability statement

The datasets presented in this study can be found in online repositories. The names of the repository/repositories and accession number(s) can be found below: https://www.ncbi.nlm.nih.gov/geo/, accession numbers GSE224549 and GSE32591.

## Ethics statement

The studies involving human participants were reviewed and approved by University of Michigan Institutional Review Board IRBMED. The patients/participants provided their written informed consent to participate in this study. The animal study was reviewed and approved by University of Michigan Institutional Animal Care and Use Committee.

## Author contributions

Conceptualization: SL and JMK; methodology: SL, CB, LT, and JMK; investigation: SL, JL, and MG-K; formal analysis: SL, JL, CB, LT, and JMK; funding acquisition: JMK; project administration: JMK; resources: JMK and JG; supervision: JMK; writing—original draft: SL, CB, LT, and JMK; writing—review and editing: SL, JL, CB, LT, MG-K, JG, and JMK. All authors contributed to the article and approved the submitted version.
