# Supplementary material for: Loss of interleukin-1 beta is not protective in the lupus-prone NZM2328 mouse model
Source: Front Immunol. 2023 May 16;14:1162799. doi: 10.3389/fimmu.2023.1162799 (PMC10227599; doi:10.3389/fimmu.2023.1162799)
Supplement: Supplementary file 1 [file DataSheet_1.pdf]

Supplementary Material

Loss of interleukin-1 beta is not protective in the lupus-prone NZM2328 mouse model

Shannon N. Loftus[1,2], Jianhua Liu[1], Celine C. Berthier[3], Johann E. Gudjonsson[4], Mehrnaz Gharaee-Kermani[1,4], Lam C. Tsoi[4,5,6], J. Michelle Kahlenberg[1,4]

[1]Division of Rheumatology, Department of Internal Medicine, University of Michigan, Ann Arbor, MI, 48109

[2]Graduate Program in Immunology, University of Michigan, Ann Arbor, MI 48109

[3]Division of Nephrology, Department of Internal Medicine, University of Michigan, Ann Arbor, MI 48109

[4]Department of Dermatology, University of Michigan, Ann Arbor, MI 48109

[5]Department of Computational Medicine and Bioinformatics, University of Michigan, Ann Arbor, MI 48109

[6]Department of Biostatistics, University of Michigan, Ann Arbor, MI 48109

*** Correspondence:**
J. Michelle Kahlenberg
mkahlenb@med.umich.edu

1 Supplementary Figures and Tables

1.1 Supplementary Figures

Supplementary Figure 1

Supplementary Figure 2

Supplementary Figure 3

1.2 Supplementary Tables

Supplementary Table 1

Supplementary Table 2

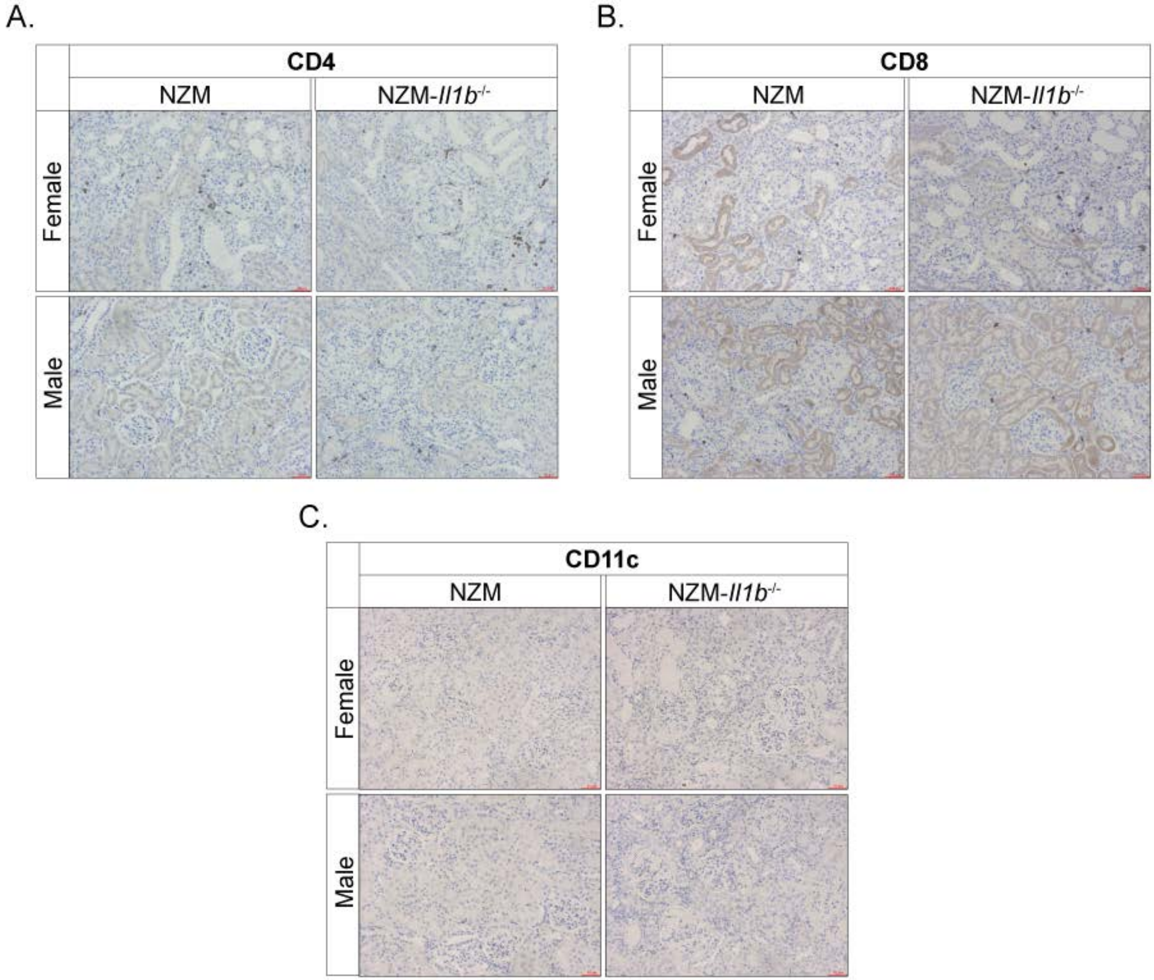

Supplementary Figure 1. Representative images of kidneys stained with (**A**) CD4, (**B**) CD8, and (**C**) CD11c.

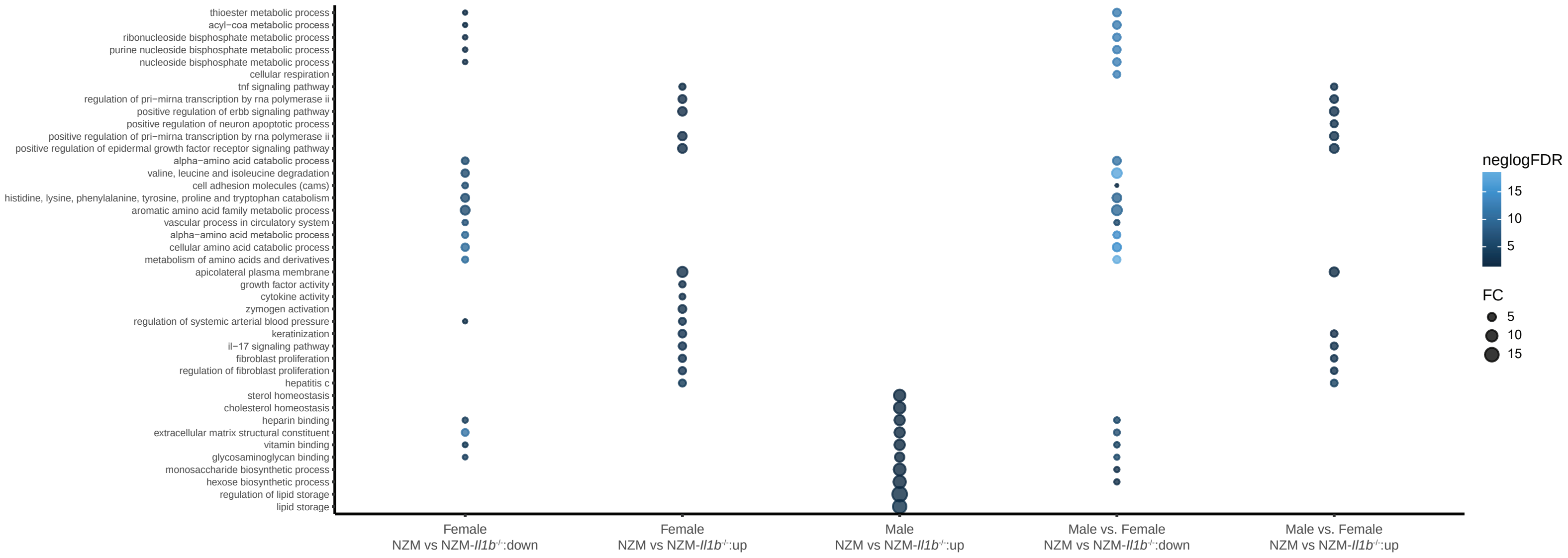

Supplementary Figure 2. Pathway analysis of differentially expressed genes between NZM and NZM-*Il1b*$^{-/-}$ kidneys highlighted the TNF (p=7.59x10^{-5}) and IL-17 signaling (p=1.21x10^{-6}) pathways among the top regulated pathways.

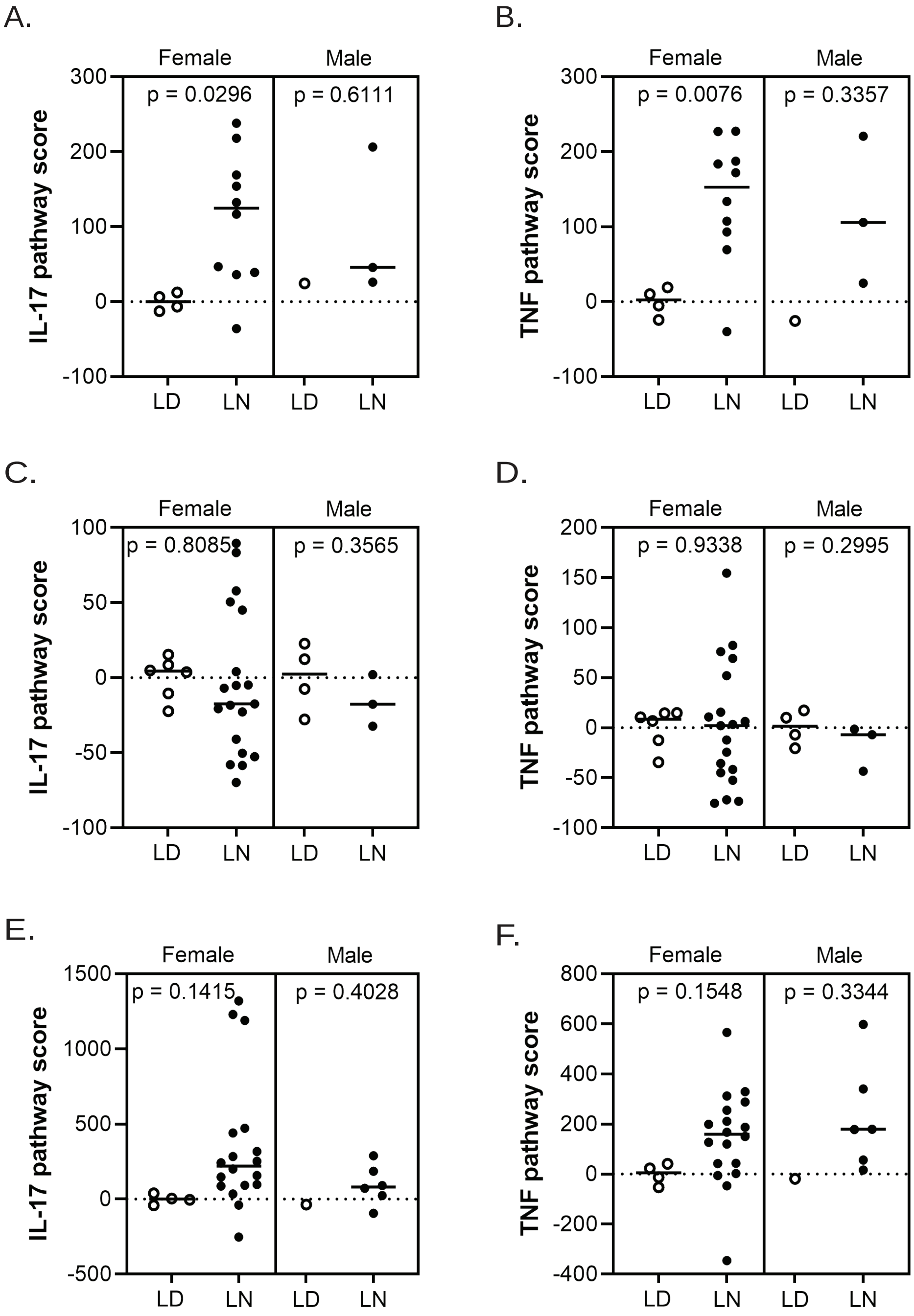

Supplementary Figure 3. RNA-sequencing gene expression analysis of **(A)** IL-17 and **(B)** TNF pathway scores in the glomerular compartment of human lupus nephritis (LN) and healthy living donor (LD) biopsies: IL-17 and TNF signaling pathway scores were significantly higher in female LN (n=10) compared to LD controls (n=4) (p=0.0296 and 0.0076 respectively), but not in males (n=1 LD and 3 LN) (p=0.6111 and 0.3357 respectively). Microarray gene expression analysis of **(C)** IL-17 and **(D)** TNF pathway scores in the tubulointerstitial compartment of human LN and LD biopsies: IL-17 and TNF signaling pathway scores were not significantly different in female LN (n=19) compared to LD controls (n=6) (0.8085 and 0.9338, respectively) or in males (n=4 LD and n=3 LN) (p=0.3565 and 0.2995, respectively). RNA-sequencing gene expression analysis of **(E)** IL-17 and **(F)** TNF pathway scores in the tubulointerstitial compartment of human LN and LD biopsies: IL-17 and TNF signaling pathway scores were not significantly different in female LN (n=18) compared to LD controls (n=4) (p=0.1415 and 0.1548, respectively) or in males (n=1 LD and 6 LN) (p=0.4028 and 0.3344, respectively).
