# Supplementary material for: Loss of interleukin-1 beta is not protective in the lupus-prone NZM2328 mouse model
Source: Front Immunol. 2023 May 16;14:1162799. doi: 10.3389/fimmu.2023.1162799 (PMC10227599; doi:10.3389/fimmu.2023.1162799)
Supplement: Supplementary file 3 [file Table_2.pdf]

Table S2: Clinical characteristics of reference biopsies analyzed by Affymetrix microarray gene expression profiling. All patients are Caucasian. Data are presented as mean ± SEM. NA: non available. * Reference: Levey AS et al. A new equation to estimate glomerular filtration rate. Ann Intern Med. 2009;150(9):604-12 (PMID: 19414839).

	**Characteristic**	**Living donors (LD) female**	**Living donors (LD) male**	**Lupus nephritis (LN) female**	**Lupus nephritis (LN) male**
Glomerular compartment	Patient number	4	9	19	3
	Age (years)	61.0 ± 3.2	48.4 ± 3.9	32.7 ± 2.3	43.9 ± 14.7
	Serum creatinine (mg/dL)	NA	1.1 ± 0.1	1.7 ± 0.2	3.0 ± 0.0
	Proteinuria (g/day)	absent	absent	3.9 ± 0.9	1.3 ± 0.5
	CKD-Epi GFR (ml/min/1.73m^2)*	NA	79.0 ± 7.7	55.6 ± 7.7	73.0 ± 9.8
Tubulointerstitial compartment	Patient number	6	4	19	3
	Age (years)	59.2 ± 3.0	42.5 ± 6.2	32.7 ± 2.3	43.9 ± 14.7
	Serum creatinine (mg/dL)	NA	1.0 ± 0.1	1.7 ± 0.2	3.0 ± 0.0
	Proteinuria (g/day)	absent	absent	3.9 ± 0.9	1.3 ± 0.5
	CKD-Epi GFR (ml/min/1.73m^2)*	NA	88.1 ± 12.4	55.6 ± 7.7	73.0 ± 9.8

Table S3: Clinical characteristics of reference biopsies analyzed by RNA-sequencing gene expression profiling. All patients are Caucasian. Data are presented as mean ± SEM. NA: non available. * Reference: Levey AS et al. A new equation to estimate glomerular filtration rate. Ann Intern Med. 2009;150(9):604-12 (PMID: 19414839).

	**Characteristic**	**Living donors (LD) female**	**Living donors (LD) male**	**Lupus nephritis (LN) female**	**Lupus nephritis (LN) male**
Glomerular compartment	Patient number	4	1	10	3
	Age (years)	50.4 ± 4.6	30.6	32.8 ± 4.9	46.9 ± 14.3
	Serum creatinine (mg/dL)	NA	NA	1.2 ± 0.2	1.0 ± 0.2
	Proteinuria (g/day)	absent	absent	5.2 ± 1.9	NA
	CKD-Epi GFR (ml/min/1.73m^2)*	NA	NA	69.4 ± 10.0	81.1 ± 15.7
Tubulointerstitial compartment	Patient number	4	1	18	6
	Age (years)	50.4 ± 4.6	30.6	37.1 ± 3.3	37.8 ± 8.1
	Serum creatinine (mg/dL)	NA	NA	1.3 ± 0.2	1.2 ± 0.2
	Proteinuria (g/day)	absent	absent	4 ± 1.1	3.4 ± 1.7
	CKD-Epi GFR (ml/min/1.73m^2)*	NA	NA	69.1 ± 8.9	81.3 ± 13.4
